# Nutritional Problems Among Special Needs Children in a Rural Special Needs Children Home Near Chennai

**DOI:** 10.7759/cureus.58236

**Published:** 2024-04-14

**Authors:** Vigneshwar K S, Pankaj B Shah

**Affiliations:** 1 Department of Community Medicine, Sri Ramachandra Institute of Higher Education and Research, Chennai, IND

**Keywords:** obesity and overweight, underweight, children with special needs, malnourishment, nutritional deficiency

## Abstract

Background

Special needs children have various health problems, and the most common problems are nutritional deficiency and malnourishment which leads to increased morbidity affecting their quality of life. This study aims to assess the nutritional status and health-seeking behaviour of special needs children.

Methods

The study was conducted among 46 special needs children at a special needs children's home. After collecting basic sociodemographic details, they were assessed for nutritional status and health-seeking behaviour using a semi-structured questionnaire followed by general and clinical examination.

Results

Out of these 46 special needs children, 69.6% were male and 30.4% were female. The mean age was 11.69±4.62 years. In this study, 65.22% were underweight; 6.52% were overweight; 10.87% were obese I; 4.35% were obese III; 13.04% were normal. Among them, 37% seek Government healthcare facilities for their healthcare needs. Referral advice was provided to all required children.

Conclusion

A high proportion of the special needs children were screened positive for nutritional deficiency and malnourishment which needs to be addressed. Interventions should be aimed at correcting the nutritional deficiency and malnourishment by involving the caretakers, mainly mothers of these children.

## Introduction

The age group of 2 to 17 years is the most important period for growth and development in all aspects for children. However unfortunately certain children lack them, specially labelled as “Special needs children” or “Children with special healthcare needs” [1,2]. They have problems in growth and development in terms of physical, developmental, behavioural and emotional conditions. They require increased attention and care and are at increased trend of seeking healthcare services like frequent hospital visits, and hospital admissions for health-related ailments [2,3]. 

The normal growth and development of children depend on their nutritional intake. Mothers are the primary caretakers, taking care of the nutritional needs of their children. It requires immense attention and additional care while looking after special needs children because they cannot express their hunger and satiety like normal children. The knowledge and understanding of the mothers about the special needs children will be beneficial in taking care of them. Lack of knowledge of the mothers about nutrition affects the health of their child leading to nutritional deficiency and malnutrition [4-7].

Health-seeking behaviour is a decision-making process to seek perfect treatment for health. It is governed by multiple factors. Special needs children are at increased risk of having unmet health needs when compared to normal children. Health-seeking behaviour has emerged as a tool to tackle ill health by taking remedial actions and people are being encouraged to learn and use health-promoting behaviours [8-11].

There are a lot of studies addressing the nutritional problems of normal children but only limited studies were done among special needs children. To bridge the knowledge gap, this is one of few studies conducted among special needs children and the main purpose of this study is to assess the nutritional problems and health-seeking behaviour of special needs children [12-14].

## Materials and methods

This cross-sectional study was conducted among special needs children attending a special children home at the Rural health training centre field practice area of a tertiary care teaching hospital (Sri Ramachandra Medical College & Research Institute) in Tiruvallur district during August and September 2022. There were a total of 46 children, clinically confirmed and certified as special needs children attending the special children home. All the special needs children were included as study participants.

Ethical considerations

The study was initiated after obtaining Ethics approval from the Institutional Ethics Committee (IEC NO: CSP-MED/22/AUG/79/130 dated 16/09/2022). Permission was obtained from the special children's home for conducting the study. Informed consent was obtained from the parents/guardians of the special needs children. The information collected from the study participants was kept confidential.

Data collection method

Data was collected from the parents/caregivers of the special needs children using a semi-structured questionnaire consisting of three parts (Part A, B & C). Part A consists of questions related to basic sociodemographic characteristics such as age, gender, birth order, number of siblings of the special needs children, type of marriage, type of family, education & occupation of both father and mother, monthly income and socioeconomic status of the family. Part B consists of questions related to nutritional statuses such as eat normally or not, symptoms of decreased eating, any illness, fever, respiratory & gastrointestinal symptoms in the past two weeks, the habit of nail biting, eating soil/pica, difficulty in concentration, symptoms of memory loss, intake of nutritional supplements or any other medications, diet history using 24-hour recall method, total calorie intake, calorie requirement and calorie deficit. Part C consists of questions related to their health-seeking behaviour for general ailments or sickness/respiratory/gastrointestinal/neurological problems such as whether sought health care services, where do you seek health care services - government hospital / private clinic/pharmacy/others, reason for not taking treatment - able to manage / not aware / not affordable / others. The special needs children were examined clinically including anthropometric measurements like height, weight, BMI, head circumference, chest circumference and mid-arm circumference.

Statistics

The data was collected and entered using Microsoft Excel. Statistical analysis was done using IBM SPSS Statistics for Windows, Version 16 (Released 2007; IBM Corp., Armonk, New York, United States). and the results were tabulated. A Chi-square test was used to find the association between nutritional problems and related variables. The level of significance was kept as a p-value < 0.05.

## Results

Sociodemographic characteristics

In this study, out of the 46 special needs children who participated, 69.6% were male and 30.4% were female. The mean age was 11.69 ±- 4.62 years with 82.6% belonging to 6 - 17 years of age. There were a total of 46 special needs children who belonged to a nuclear family and they lived in a rural area. The parents of all the children were educated and the fathers were employed, with 71.7% belonging to Socioeconomic Class III according to Modified B.G. Prasad Classification (Table 1).

**Table 1:** Sociodemographic characteristics of the study participants The data have been represented as frequency(n) and percentage (%). The socioeconomic status was classified based on Modified B.G. Prasad Classification 2023

Variables	Frequency (n)	Percentage (%)
Age		
2 to 5 years	8	17.4%
6 to 17 years	38	82.6%
Gender		
Male	32	69.6%
Female	14	30.4%
Birth Order		
First	29	63%
Second	16	34.8%
Third	1	2.2%
Type of Marriage		
Consanguinous	21	45.7%
Non-consanguinous	25	54.3%
Father’s Education		
Primary	8	17.4%
Middle	24	52.2%
Secondary	11	23.9%
Graduate & above	3	6.5%
Mother’s Education		
Primary	4	8.7%
Middle	23	50%
Secondary	13	28.3%
Graduate & above	6	13%
Mother’s Occupation		
Working	24	52.2%
Not working	22	47.8%
Monthly Income		
0 to 10,000	2	4.3%
10,001 to 20,000	36	78.3%
20,001 to 30,000	6	13%
30,001 to 40,000	2	4.3%
Socio Economic Status		
Class I	-	-
Class II	7	15.2%
Class III	33	71.7%
Class IV	6	13%
Class V	-	-
Total Family Size		
< 5	14	30.4%
> 5	32	69.6%
Number of Siblings		
< 1	41	89.1%
> 1	5	10.9%

Nutritional problems

The study revealed that 78.3% of the children have a calorie deficit of more than 300 kcal even though most of the children eat normally (97.8%). Also, 100% of the children did not have the following symptoms gastrointestinal symptoms in the past two weeks; habit of nail biting; difficulty in concentration; symptoms of memory loss; intake of nutritional supplements and other symptoms. Only a few children had symptoms of decreased eating, illness, fever and respiratory symptoms in the past two weeks and habit of eating soil/pica (Table 2).

**Table 2:** Nutritional problems among the study participants The data are represented as frequency(n) and percentage (%)

Variables	Frequency (n)	Percentage (%)
Does the child eat normally?		
Yes	45	97.8%
No	1	2.2%
Does the child have symptoms of decreased eating?		
Yes	4	8.7%
No	42	91.3%
Did the child have any illness in the past two weeks?		
Yes	10	21.7%
No	36	78.3%
Did the child have fever in the past two weeks?		
Yes	1	2.2%
No	45	97.8%
Did the child have any respiratory symptoms like cough, runny nose, nasal congestion & sore throat in the past 2 weeks?		
Yes	10	21.7%
No	36	78.3%
Does the child have the habit of eating soil/pica?		
Yes	2	4.3%
No	44	95.7%
Does the child take any other medications?		
Yes	2	4.3%
No	44	95.7%
Calorie deficit		
Yes	36	78.3%
No	10	21.7%

Nutritional status

Based on the anthropometric measurements, the nutritional status of the special needs children revealed that 65.22% were underweight and only 13.04% were found to be of normal weight (Table 3).

**Table 3:** Nutritional status of the study participants based on examination The data have been represented as frequency(n) and percentage (%). The nutritional status was classified based on WHO BMI Classification.

Nutritional Status	Frequency (n)	Participants (%)
Underweight	30	65.22 %
Normal Weight	6	13.04 %
Obese I	5	10.87 %
Overweight	3	6.52 %
Obese III	2	4.35%
Total	46	100%

Health-seeking behaviour

Assessment of the health-seeking behaviour of the participants revealed that 63% of them prefer private healthcare facilities and 37% of them seek Government healthcare facilities for their healthcare needs.

Association of nutritional problems with related variables

This study revealed that the association of nutritional problems with related variables using the Chi-Square test shows a significant association of nutritional problems with calorie deficit and monthly income, whereas the rest of the variables were found to be not significant (Table 4).

**Table 4:** Association between nutritional problems and related variables The data have been represented as frequency(n) and percentage (%). χ2 = Chi-square value is considered significant between <2 z-score and > −3 z-score. The p-value is considered statistically significant at p < 0.05

Variables	Nutritional Problems				Chi-square value	Odd’s Ratio	95% C.I.	p-Value
	Present		Absent					
	n (30)	%	n (16)	%				
Age								
2 to 5 years	6	75%	2	25%	0.409	1.750	0.310 – 9.878	0.523
6 to 17 years	24	63.2%	14	36.8%				
Gender								
Female	9	64.3%	5	35.7%	0.008	0.943	0.253 – 3.509	0.930
Male	21	19.6%	11	10.9%				
Birth Order								
Second & above	13	76.5%	4	23.5%	1.505	2.294	0.599 – 8.782	0.220
First	17	58.6%	12	41.4%				
Mother’s Occupation								
Working	14	58.3%	10	41.7%	1.048	0.525	0.152 – 1.815	0.306
Not Working	16	72.7%	6	27.3%				
Monthly Income								
< 20,000	28	73.7%	10	26.3%	6.905	8.400	1.451 – 48.613	0.009*
> 20,000	2	25%	6	75%				
Socioeconomic status								
Low	4	66.7%	2	33.3%	0.006	0.929	0.151 – 5.717	0.936
High	26	65%	14	35%				
Total Family Size								
< 5	21	65.6%	11	34.4%	0.008	1.061	0.285 – 3.948	0.930
> 5	9	64.3%	5	35.7%				
No. of. Siblings								
< 1	3	60%	2	40%	0.067	0.778	0.116 – 5.211	0.795
> 1	27	65.9%	14	34.1%				
Decreased Eating								
Yes	3	75%	1	25%	0.185	1.667	0.159 – 17.468	0.667
No	27	64.3%	15	35.7%				
Illness								
Yes	8	80%	2	20%	1.231	2.545	0.471 – 13.770	0.267
No	22	61.1%	14	38.9%				
Respiratory Symptoms								
Yes	8	80%	2	20%	1.231	2.545	0.471 – 13.770	0.267
No	22	61.1%	14	38.9%				
Eat Soil/Pica								
Yes	1	50%	1	50%	0.213	0.517	0.030 – 8.862	0.644
No	29	65.9%	15	34.1%				
Calorie Deficit								
> 300 kcal	27	81.8%	6	18.2%	14.186	15.000	3.138 – 71.693	0.000*
< 300 kcal	3	23.1%	10	76.9%				

## Discussion

Out of the 46 special needs children assessed, the study revealed that 65.22% were underweight and malnourished which is high when compared to normal children. Only few studies were done previously in special needs children. In India, a study conducted by Katoch found that 19% of children were underweight [15]. Similarly, in Indonesia, a study by Kementerian Kesehatan found that 17.1% of children under 5 years of age were underweight, while another study by Wungouw et al. in Indonesia reported that 15.3% of children were underweight [16,17]. In Ethiopia, a study by Simon et al. revealed that 42% of children were underweight, which is higher than the global prevalence of 22.5% [18].

In children, adequate food intake and nutrition are essential to achieve their normal growth and development. In our study, 78.3% of children were found to have a calorie deficit of more than 300 kcal causing malnourishment which is comparable to a study done in Bali by Permatananda et al. where 68.3% of children have less nutrition intake compared to children with adequate nutrition intake [19]. The reasons for high-calorie deficit in our study were low monthly income and lack of awareness of balanced nutrition. Balanced and adequate nutrition is important to improve the quality of life of children [7]. The nutritional requirements of the special needs children were not met due to economic circumstances as the primary caretaker has to be with them all day, not allowing them to go to work. Malnourishment is commonly seen due to the economic burden of the family. Malnutrition makes the children vulnerable to infections and weight loss which can lead to complications, increased morbidity and mortality. The lack of knowledge about nutrition among the primary caretakers also affects the growth and development of the children [6,7].

According to Mkhize, household food insecurity and low household income are the leading factors affecting the nutritional status of children under five years of age in South Africa. Underweight and overweight are the nutritional indicators used to evaluate and monitor the nutritional status of the children. The nutritional status of the children in developed countries is not majorly affected by poverty or economic growth but rather by eating disorders. Frequent and prolonged illness in children can also affect their nutritional status, leading to changes in appetite, absorption, metabolism, and behaviour. Conversely, poor nutritional status can make the children more prone to illness or lengthen their recovery time [4,6].

According to the results of our study, only calorie deficit and monthly income show significant association with nutritional problems which states that low-income people take less nutrition. In contrast to this statement, the Katoch study states that in India, low-income people consume more calories with less malnutrition rates as they are covered by nutrition schemes of the government like Public Distribution System and Integrated Child Development Services providing quality nutrition foods and awareness of balanced nutrition [15]. The programs covering these special needs children under Maternal and Child Health (MCH) and Children With Special Health Care Needs (CSHCN) were responsible for planning and developing the health care systems and providing health care services to them [1,20]. This study enlightens the nutritional problems of the special needs children necessitating actions to be taken to improve their quality of life. 

Our study had some limitations that need to be acknowledged. Firstly, we included special needs children from one home in this study which in future studies can include different homes to obtain a more comprehensive conclusion. Secondly, we only used anthropometry and clinical examination for our analysis and in future studies, we can include biochemical laboratory investigations.

## Conclusions

This study revealed that a high proportion of the special needs children were underweight. The most important reasons for being underweight were found to be calorie deficit, low monthly income of the family, and lack of awareness of balanced nutrition among the caretakers. Most of the mothers who were the primary caretakers of these children were unable to go for work which increases the economic burden of the family. Increasing parental awareness and emphasizing the importance of balanced nutrition with cost-effective, seasonal, and locally available nutritious foods can help the caretakers manage food properly. It further helps them to meet the nutritional requirements and ensures the normal growth and development of the special needs children.

## Appendices

**Table 5:** PART – A: Questions related to Sociodemographic characteristics

Age	2 to 5 years					6 to 17 years				
Gender	Male					Female				
Birth Order	First			Second				Third		
Type of Family	Nuclear			Joint				Others		
Locality	Rural					Urban				
Type of Marriage	Consanguinous					Non-Consanguinous				
Father’s Education	Illiterate	Primary			Middle		Secondary			Graduate
Father’s Occupation	Working					Not Working				
Mother’s Education	Illiterate	Primary			Middle		Secondary			Graduate
Mother’s Occupation	Working					Not Working				
Monthly Income	0 - 10000		10001 - 20000			20001 - 30000			30001 - 40000	
Socio Economic Status	Class I	Class II			Class III		Class IV			Class V
Total Family Size	< 5					> 5				
Number of Siblings	< 1					> 1				

**Table 6:** PART – B: Questions related to nutritional status

Does the child eat normally?	Yes	No
Does the child has symptoms of decreased eating?	Yes	No
Did the child had any illness in the past 2 weeks?	Yes	No
Did the child had fever in the past 2 weeks?	Yes	No
Did the child had any respiratory symptoms like cough, runny nose, nasal congestion & sore throat in the past 2 weeks?	Yes	No
Did the child had any gastrointestinal symptoms like abdominal pain, constipation & diarrhoea in the past 2 weeks?	Yes	No
Does the child has the habit of nail biting?	Yes	No
Does the child has the habit of eating soil/pica?	Yes	No
Does the child has difficulty in concentration?	Yes	No
Does the child has symptoms of memory loss?	Yes	No
Does the child takes nutritional supplements?	Yes	No
Does the child takes any other medications?	Yes	No
Does the child has any other symptoms?	Yes	No
Total Calorie Intake		
Calorie requirement		
Calorie deficit		

## References

[1] McPherson M, Arango P, Fox H (1998). A new definition of children with special health care needs. Pediatrics.

[2] Avis M, Reardon R (2008). Understanding the views of parents of children with special needs about the nursing care their child receives when in hospital: a qualitative study. J Child Health Care.

[3] Newacheck PW, Strickland B, Shonkoff JP (1998). An epidemiologic profile of children with special health care needs. Pediatrics.

[4] Mkhize M, Sibanda M (2020). A review of selected studies on the factors associated with the nutrition status of children under the age of five years in South Africa. Int J Environ Res Public Health.

[5] Mulyani NS, Arnisam A, Andriani A, Fitrianingsih E, Hadi A, Al Rahmad AH (2023). The effect of nutrition counseling on mother’s knowledge and nutrition information in autistic children in Banda Aceh City. Open Access Maced J Med Sci.

[6] Almulla AA, Alanazi AS, Khasawneh MA (2023). The relationship between nutritional intake and mother’s education level with the nutritional status of children with special needs. J Reatt Ther Dev Divers.

[7] Aminah D, Ardiana A, Rahmawati I (2023). Nutritional improvement interventions for malnourished preschool children: a systematic review. UNEJ E-Proceeding.

[8] Burtscher D, Burza S (2015). Health-seeking behaviour and community perceptions of childhood undernutrition and a community management of acute malnutrition (CMAM) programme in rural Bihar, India: a qualitative study. Public Health Nutr.

[9] Chandwani H, Pandor J (2015). Healthcare-seeking behaviors of mothers regarding their children in a tribal community of Gujarat, India. Electron Physician.

[10] Ngangbam S, Roy AK (2019). Determinants of health-seeking behaviour in Northeast India. J Health Manag.

[11] Chauhan RC, Manikandan PA, Samuel A, Singh Z (2015). Determinants of health care seeking behavior among rural population of a coastal area in South India. Int J Sci Rep.

[12] Islary J (2014). Health and health seeking behaviour among tribal communities in India: a socio-cultural perspective. J Tribal Intellect Collect India.

[13] Barua K, Borah M, Deka C, Kakati R (2017). Morbidity pattern and health-seeking behavior of elderly in urban slums: a cross-sectional study in Assam, India. J Family Med Prim Care.

[14] Chaturvedi S, Sharma N, Kakkar M (2017). Perceptions, practices and health seeking behaviour constrain JE/AES interventions in high endemic district of North India. BMC Public Health.

[15] Katoch OR (20241). Tackling child malnutrition and food security: assessing progress, challenges, and policies in achieving SDG 2 in India. Nutr Food Sci.

[16] Munira SL (2023). Hasil Survei Status Gizi Indonesia (SSGI) 2022..

[17] Wungouw H, Ottay R, Rumampuk H, Rumampuk I, Pangemanan M (2023). Nutritional status of children at Kaima village, North Minahasa Regency, Indonesia. AIP Conf Proc.

[18] Olcina Simón MA, Soriano JM, Morales-Suarez-Varela M (2023). Assessment of malnutrition among children presenting in a nutrition center in Gimbichu, Ethiopia. Children (Basel).

[19] Permatananda PANK, Pandit GS (2023). Nutritional status of children age 4-6 years old in local village. J Penelit Pendidik IPA.

[20] Alur M (2002). Special needs policy in India. Educ Child Spec Needs Segreg Incl New Delhi Sage Publ.

